# Exploring intentions: factors influencing international study decisions in healthcare bachelor degree programs

**DOI:** 10.1186/s12909-025-07136-4

**Published:** 2025-04-17

**Authors:** Ann Kristin Bjørnnes, Astrid Torbjørnsen, Björg Sigridur Anna Thordardottir, Anne Lund, Olav Johannes Hovland, Lisebet Skeie Skarpaas

**Affiliations:** Faculty of health science, Institute of Nursing and Health Promotion, Oslo Metropolitan University, St. Olavs plass, P.O. Box 4, Oslo, NO-0130 Norway

**Keywords:** Exchange, Health care students, International studies, Higher education, Internationalization, International students, Questionnaires, Barriers and constrains

## Abstract

**Background:**

Study abroad programs offer significant benefits for healthcare students, including enhanced cultural awareness and professional development. However, participation in these programs is often influenced by various enablers and barriers. Understanding students’ characteristics, preferences, and knowledge about exchange opportunities is important for supporting students and developing strategies to facilitate exchange.

**Methods:**

This study conducted a cross-sectional survey among students enrolled in four 3-year professional health bachelor’s programs in Norway. It aimed to investigate students’ characteristics, preferences, and knowledge about exchange opportunities. Additionally, it sought to examine enablers and barriers to exchange, as well as associations between students’ characteristics and their intention to participate in exchange programs.

**Results:**

The survey results (*N* = 192) suggest associations between social relationships, financial considerations, personal motivations, and students’ decisions regarding participation in exchange programs.

**Conclusion:**

This study provides a more nuanced understanding of students’ characteristics, preferences, and barriers associated with the intention to participate in international exchange opportunities within health science programs. Institutions should address the practical and personal challenges that students may encounter, thereby ensuring equitable access and participation. Furthermore, the study offers insights into the initial stages of the exchange process, focusing not only on those who choose to participate in exchange programs but also on those who opt out.

**Supplementary Information:**

The online version contains supplementary material available at 10.1186/s12909-025-07136-4.

## Background

Internationalization has become a key change agent in higher education, driven by globalization and the knowledge economy [1]. Several international studies have demonstrated the positive impact of international placements on students, indicating significant benefits for both personal and professional development [2]. For healthcare students, taking part in international study abroad programs enhance cultural awareness and provides opportunities for professional development [3–6]. Furthermore, international placements provide allied health students with unique learning outcomes, such as personal and professional development, cultural insights, and understanding of diverse healthcare contexts [7, 8]. Students have reported that exchange programs helped them accept diversity, collaborate across boundaries, and gain confidence from challenges [6]. These benefits persisted over time and into their professional careers [6, 9]. Despite extensive documentation of immediate results, research on long-term impact and the characteristics of students who apply for, as well as those who do not apply for, these programs remain limited [10, 11].

A meta synthesis suggests that similar factors across various locations and professional groups influence healthcare students’ decisions to participate in international studies. These include adequate early information about the programs, interest in other cultures, and positive role models from academic staff and family encouraging studies abroad [3]. A main barrier to going on exchange has previously been reported to be financial constraints [3, 12], and cost issues are a significant concern across countries [13]. Another important barrier is language proficiency. Even with bursaries like the Erasmus scheme, language can be an issue if students aren’t fluent in the host country’s language [3]. Barriers include also personal commitments, and safety concerns [4]. In addition, recognition of study credits and socio-economic background has also been reported to influence participation [13].

In the Nordic context, the choice of international mobility for students is influenced not just by the educational quality or the reputation of the host university. It also takes into account the appealing aspects of the country they are moving to [14]. Exchange programs, both short-term and long-term, have been positively evaluated by students [10, 15, 16]. These programs serve to expand the students’ perspectives [17], which, in turn, reinforces their resolve to pursue their degree and to stay committed to the program [18, 19]. To provide culturally diverse experiences and stimulate interest in future exchange opportunities, innovative approaches like Internationalization at Home are being employed. These initiatives, such as utilizing technology for cross-cultural learning sessions, are designed to equip students for global practice while overcoming barriers associated with traditional international experiences [20]. Incorporating virtual exchange into curriculum design can enhance intercultural competence, thereby preparing healthcare students for their future professional roles [21, 22]. In Norway, students have acknowledged the valuable insights they acquired from their global partners through participating in a Collaborative Online International Learning (COIL). These insights are directly applicable to the care of marginalized populations within Norway [23], and may serve to motivate students for future exchange programs.

The government in Norway has an ambition that at least 50% of the students in higher education should participate in long-term mobility at least once during their studies [24]. This enhanced focus on internationalization is not only a Norwegian phenomenon but are emphasized in a European and Global context as well. Global citizenship is valued as an important goal to meet challenges related to health, diversity, innovation, technology and occupational justice. Internationalization is no longer a primarily Western concept. Globally, institutions are engaging in this process, considering its impact on policy and practice. In the broader context, internationalization is not merely a goal but a means to enhance the quality of education, research, and societal service [25]. Although during and after the pandemic, our experience has been that internationalization has declined rather than expanded.

Given the relatively small proportion of allied health students participating in study abroad programs, it becomes essential to address the issue of equitable access to these international experiences.

There is currently a renewed focus on studying barriers to internationalization due to the decline in international activities during the pandemic. In addition, other factors may also contribute to barriers to studying abroad, such as the financial need for most Norwegian students to have an income while studying (84%) [26], and the high prevalence of mental health issues among students [27]. There is a noticeable gap in understanding the experiences of students before they apply, particularly those who are interested but may lack the resources to take part [11]. Therefore, it is important to investigate these experiences during the pre-application phase. Understanding these preliminary experiences could provide valuable insights into the motivations and potential obstacles for allied health students considering international mobility programs.

This study aims to investigate students’ characteristics, preferences, and knowledge about exchange opportunities in 3-year professional health science bachelor programs. It also seeks to examine enablers and potential barriers to exchange, as well as associations between students’ characteristics and their intention to participate in exchanges during the program.

## Methods

### Context

The study is conducted within the Faculty of Health Sciences at Oslo Metropolitan University, Norway, which accommodates approximately 6 000 students across five departments. Notably, the Faculty hosts the largest bachelor’s programs in nursing and occupational therapy in Norway. Other programs included in this study are the social nurse educational program and the paramedic educational program. These four programs have received funding from the Norwegian Directorate for Higher Education and Skills (HkDir) program, titled “Increased student mobility in 3-year bachelor’s degree program.” In Norway, all health program plans adhere to the National Guidelines for Health and Social Sciences Education (RETHOS) [28], which emphasize curriculum flexibility to accommodate mobility opportunities during the degree.

### Procedure

The study employs a two-stage survey approach targeting both first-year and third-year students within the Faculty of Health Sciences’ bachelor programs at Oslo Metropolitan University. The first wave of the survey in May 2024, which forms the basis of this article, captures cross-sectional data on students’ characteristics, expectations, and intentions regarding student mobility. The design of the survey allows for a second wave of data collection when the first-year students reach their final year of study in 2027, aiming to update the information on their actual mobility experiences throughout their program (i.e., future work).

The survey was administered by the administrative international coordinator office, which sent out an email containing a link to a web platform for the survey. This platform provides secure storage in line with the Data Protection Services in Norway. This email was sent to the eligible participants in the Faculty of Health Sciences’ bachelor programs included in this study. There was no direct contact between the researchers and the participants. After the initial email containing the survey link was sent by the administrative international coordinator, reminders were sent out after two weeks and again after three weeks to all eligible students.

To ensure data protection and privacy, participants had the option to withdraw their consent at any point during the survey process. They could do so by notifying the administrative international coordinator through the email address provided in the survey invitation. The study was approved by the Norwegian Agency for Shared Services in Education and Research (Sikt) [29] before the survey was conducted.

### Measures

This survey investigated various factors that might influence student mobility choices within the context of 3-year health professional bachelor’s degree programs. In selecting the specific questions for this study, we sought to capture both the benefits and barriers identified in prior research on international exchange programs [4, 8, 9]. The questions included address cultural competence, personal growth, and challenges such as socioeconomic status and language barriers to understand the factors influencing students’ decisions to study internationally. In the demographic form, we captured information about age, sex, previous education, work experience, marital status, presence and ages of children, and parental education level. We also asked the students about their knowledge of exchange opportunities in the program, the best time for an exchange, which courses to take, the available monetary support system, and whether the courses were taught in English. All these questions were categorical, requiring a yes/unsure/no response. We also asked the students to rate their English skills on a 0–10 numeric scale, with higher scores indicating better skills.

The students were asked about their perceived gains from exchange through six questions on a Likert scale, ranging from 1 (strongly disagree) to 5 (strongly agree). In addition, they were asked to rate perceived barriers to exchange through eight questions on a Likert scale, with 1 being ”not at all important” and 5 being “very important”. The questions presented in this article were developed for this study and are displayed in Supplemental File 1.

The qualitative material was retrieved through an open-ended question; “Do you experience other barriers to going on exchange? Feel free to elaborate here”. Fifty of the 192 respondents had chosen to elaborate on their experienced barriers, and these answers constituted the data material that was analyzed.

### Data analysis

Quantitative data were analysed using IBM SPSS (Statistical Package for the Social Sciences) Statistics version 29. Descriptive analysis was performed for all variables collected in the survey, with only 2 missing values on the intention to exchange variable and 4 missing values on the barriers and gains variable.

A logistic regression analysis was conducted to explore which factors were associated with the dependent variable: intention to participate in an exchange program. The dependent variable was recoded to prepare for analysis (0 = No; Unsure, 1 = Yes).

Multivariate logistic regression was then performed. The variables assessed were included in a logistic regression analysis for a single model using a backward elimination procedure. All variables were incorporated in a multivariate analysis (i.e., Step 1) and subsequently removed step by step until all remaining variables were considered to make a significant contribution to the dependent variable (*p* < 0.005). The model’s goodness of fit was evaluated using the Hosmer-Lemeshow test [30].

Qualitative data was analyzed by using content analysis based on Graneheim and Lundman [31]. The text from the open questions was systematized through Excel. The text was divided into meaning units that are the constellation of words or statements that relate to the same central meaning and coded initially separately by two researchers (Table 1). The researchers discussed their initial codes, and decided upon a final coding set, and categories overarching the codes. Themes were discussed and developed. After re-coding of the whole material based on the final codebook, finalizing of categories and themes was agreed upon (Table 2). Due to the fact that the material was from a survey we decided to address the aspects that were visible and obvious referred to as manifest content analysis [31]. The themes and categories are used to describe the results, and the codes and some quotations are drawn upon to exemplify and deepen the description of the qualitative content.

**Table 1:** The analysis process. Theme and category with code and example of meaning unit

Theme	Category	Code	Example of meaning unit
Personal barriers	Health issues	Disease	I have a weakened immune system and become very sick quickly. I also have chronic fatigue syndrome which can make it difficult if the exchange takes place over a long period

**Table 2:** Overview of themes, categories and codes

Theme	Category	Codes
Prerequisites for international exchange	Housing	Housing away
		Housing at home
	Work issues	Not able to leave work at home
	Communication	Dialogue with the exchange university
	Follow-up by the university	Lack of information from university
		Lack of follow-up by the university
	Practical issues	Applying for visa
	Language	Language barriers
	University demands	Grade requirements
		University frames for exchange
	University facilitation	The educational program’s facilitation for exchange
	Economic issues	Loss of income
		Costs of travelling
		Low levels of scholarships/stipend
Personal barriers	Strain	Stress
		Worrying for practical issues
	Health issues	Disease
		Need for accommodation
	Access to employment at home	Work possibilities
	Priority of the Norwegian context	Need of experience with the Norwegian systems
	Sense of safety	To be away from home
		Outside the comfort zone
Relations	Family relations	Children
		Family
		Pets
	Social relations	Afraid to be alone
		Friends

## Results

This cross-sectional study consisted of 192 participants from four study programs: Nursing (*n* = 121), Occupational Therapy (*n* = 31), Learning Disability Nurse (*n* = 28), and Paramedic (*n* = 12) (Table 3). The majority, 82%, were women (*n* = 157), with a significant difference in sex distribution across the study programs (*p* = 0.033). Most participants were young, with 37% (*n* = 70) under the age of 22, and another 38% (*n* = 72) are between the ages of 22–25. The majority, 64% (*n* = 123), were in their first year of study, with a significant difference in the distribution across study programs (*p* = 0.001). Approximately 33% (*n* = 64) had pursued higher education previously, with no significant difference across study programs (*p* = 0.439).

Most participants were single (58%, *n* = 111) and 10% (*n* = 20) had children under 18 years. Participants’ parents’ educational levels varied, with 8% (*n* = 16) having a primary education, 33% (*n* = 63) a high school education, 33% (*n* = 64) university education for less than 4 years, and 25% (*n* = 48) university education for more than 4 years. Most participants were of Scandinavian origin (63%, *n* = 120), with no significant difference across study programs (*p* = 0.581). Nearly 19% (*n* = 37) had previous exchange experience, and the majority (83%, *n* = 160) had friends who had been on exchange.

When asked about their interest in going on an exchange, 47% (*n* = 91) expressed interest, 27% (*n* = 52) were unsure, and 25% (*n* = 47) were not interested. The distribution of these responses across the study programs was not statistically significant (*p* = 0.137).

**Table 3:** Characteristics of the participants

	Total	Nursing	Occupational Therapy	Learning Disability Nurse	Paramedic	*P* value
	192	(*n* = 121)	(*n* = 31)	(*n* = 28)	(*n* = 12)	
Sex, N (%)						
Women	157 (81.8)	105 (86.8)	26 (83.9)	20 (71.4)	6 (50)	
Men	34 (17.7)	15 (12.4)	5 (16.1)	8 (28.6)	6 (50)	0.033
Age group, N (%)						
< 22 years	70 (36.5)	46 (38)	12 (38.7)	7 (25)	5 (41.7)	
22–25	72 (37.5)	46 (38)	11 (35.5)	11 (39.3)	4 (33.3)	0.946
26–30	25 (13)	15 (12.4)	3 (9.7)	5 (17.9)	2 (16.7)	
> 30	25 (13)	14 (11.6)	5 (16.1)	5 (16.1)	1 (8.3)	
Study Year 1	123 (64.1)	82 (67.8)	24 (77.4)	11 (39.3)	6 (50)	0.001
Previous higher education, yes	64 (33.3)	36 (29.8)	11 (35.5)	11 (39.3)	6 (50)	0.439
Marital Status, Single	111 (57.8)	75 (62)	18 (58.1)	12 (42.9)	6 (50)	0.172
Children < 18 years	20 (10.4)	14 (11.6)	2 (6.5)	3 (10.7)	1 (8.3)	0.968
Parents educational level:						
Primary	16 (8.3)	12 (9.9)	0 (0)	4 (14.3)	0 (0)	
High school	63 (32.8)	32 (26.4)	11 (35.5)	14 (50)	6 (50)	0.268
University < 4	64 (33.3)	45 (37.2)	11 (35.5)	5 (17.9	3 (25)	
University > 4	48 (25)	31 (25.6)	9 (29)	5 (17.9)	3 (25)	
Origin, Nordic	120 (62.5)	75 (62)	18 (58.1)	18 (64.3)	9 (75)	0.581
Previous exchange	37 (19.3)	25 (20.7)	2 (6.5)	8 (28.6)	2 (16.7)	0.169
Friends exchanging	160 (83.3)	98 (81)	26 (83.9)	24 (85.7)	12 (100)	0.392
Want to exchange						
Yes	91 (47.4)	53 (43.8)	13 (41.9)	16 (57.1)	9 (75)	
Unsure	52 (27.1)	33 (27.3)	11 (35.5)	5 (17.9)	3 (25)	0.137
No	47 (24.5)	35 (28.9)	6 (19.4)	6 (21.4)	0 (0)	

Table 4 presents an overview of the students’ knowledge about exchange programs and their intentions to participate in them. Almost all students were aware of the exchange programs, and those intending to go on exchange were significantly more knowledgeable about when and where to go for the exchange. However, only a third of the students wished to exchange in theoretical courses, but this desire was significantly higher among those intending to go on an exchange. A similar pattern emerged for those wanting to exchange in clinical courses. Notably, the choice of exchange destination differed between students intending to go on exchange and those not intending to go, depending on whether the preferred location was offered in their program. Knowledge about ERASMUS support was significantly higher in those intending to exchange, while knowledge about financial support from The Public Administrative Body for Education in Norway (Lånekassen) was high across the board. Almost all students, regardless of their intentions, lacked knowledge about other sources of support.

Most students claimed they had not had any courses in English during the program, and there was no significant difference in this respect between the groups. However, those intending to go on exchange rated their English skills significantly higher than those not intending to exchange (*p* = 0.01).

**Table 4:** Students` knowledge about exchange dived by intention to exchange during the program

Intention to exchange	Total (*n* = 190)	No (*n* = 99)	Yes (*n* = 91)	*P*-value
Knowledge about exchange in the program (Yes)	164 (86.3)	82 (82.8)	82 (90.1)	0.322
Knowledge about when to exchange (Yes)	135 (71.1)	63 (63.6)	72 (79.1)	0.032
Knowledge about destinations (Yes)	145 (76.3)	67 (67.7)	78 (85.7)	0.003
Want to exchange in theoretical courses (Yes)	60 (31.6)	20 (20.2)	40 (44)	< 0.001
Want to exchange in clinical courses (Yes)	115 (60.5)	38 (38.4)	77 (84.6)	< 0.001
Are your preferred destination offered in your program (Yes)	74 (38.9)	22 (22.2)	52 (77.1)	< 0.001
Knowledge about ERASMUS support (Yes)	104 (54.7)	40 (40.4)	64 (70.3)	< 0.001
Knowledge about Lånekassen support (Yes)	172 (90.5)	90 (90.9)	82 (90.1)	0.523
Knowledge about other support sources (No)	187 (98.4)	97 (98)	90 (98.9)	0.532
Have one of your courses been taught in English (No)	163 (85.5)	88 (88.9)	75 (82.4)	0.143
English skills: numeric rating 0–10, mean (SD)		8.05 (2.1)	8.74 (1.4)	0.010

Table 5 presents data on perceived gains from exchange programs, with students rating the importance of various aspects on a Likert scale of 1 to 5, where 1 represents “Strongly Disagree” and 5 represents “Strongly Agree”. All students generally agreed that these were significant gains from participating in exchange programs, with both the mean and mode of 5. Table 6 presents the perceived barriers to participating in exchange programs rated on a Likert scale from 1 to 5, where 1 represents “Not at all important” and 5 represents “Very important”. The ratings varied across the different barriers, with renting out their apartment and loss of income being perceived as very important, while potential job loss was considered only slightly important.

**Table 5:** Perceived gains from exchange (Range 1 to 5)

Perceived gains (*n* = 188)	Median	Mode
Learning about communication	5	5
Learning about culture	5	5
Improve language skills	5	5
Learning new skills	5	5
Improved creative skills	5	5
Improved flexibility	5	5

1 = Strongly Disagree, 2 = Disagree, 3 = Neither Agree nor Disagree, 4 = Agree, 5 = Strongly Agree

**Table 6:** Perceived barriers to exchange (Range 1 to 5)

Barriers to exchange (*n* = 188)	Median	Mode
To rent out my apartment	4	5
Losing my job if I go on exchange	2	1
Losing income	4	5
Clinical placement in Norway is important for summer work	3	3
Clinical placement in Norway is important for post graduate work	3	3
Language barriers	3	4
Too much work to prepare for exchange	4	4
The application process is too extensive	4	4

1 = Not at all important, 2 = Slightly important, 3 = Neutral, 4 = Moderately important, 5 = Very important

In the logistic regression analysis (Table 7), several factors were considered at Step 1. These include age, sex, field of study (with Learning Disability Nurse as the reference category), marital status, having children under 18 at home, parents’ level of education, English language skills rated from 0 to 10, having taken higher education, origin, previous exchange experience, having friends who have been on exchange, and perceived barriers to exchange sum score. By Step 9, the factors that appeared to influence the intention to go on exchange were study program (i.e., nursing), marital status, English language skills, and previous exchange experience.

Nursing students showed approximately 4.72 times higher odds of considering going on an exchange compared to the reference category. Among the entire group of students, those who are single appeared more likely to intend to go on an exchange than those in a relationship (OR = 0.313, *p* < 0.001). Each unit increase in English skills, on a scale of 0–10, was associated with a 1.28 times higher likelihood of intending to go on exchange (*p* = 0.015). Additionally, students with previous exchange experience showed higher odds of intending to go on exchange, with an odds ratio of 4.66 (*p* < 0.001). This suggests a positive association between previous exchange experience and the intention to go on exchange. Other factors, including age and sex, did not show a statistically significant association with the intention to go on exchange by Step 9.

**Table 7:** Logistic regression variables associated with intention to exchange during the study program (*N* = 190)

Step	Variable	B	Sig.	Exp(B).	95% C.I. for EXP(B) Lower	95% C.I. for EXP(B) Upper
1*	Age (> 30 year as reference)		0.868			
	> 22 years	0.006	0.989	1.006	0.435	2.328
	22–25 years	− 0.127	0.827	0.880	0.282	2.750
	26–30	− 0.613	0.425	0.542	0.120	2.443
	Sex (female/male)	− 0.513	0.304	0.598	0.225	1.593
1	Study program (Learning Disability Nurse as reference)		0.081			
	Nursing	1.809	0.026	6.103	1.237	30.117
	Paramedic	0.029	0.952	1.029	0.406	2.608
	Occupational therapy	0.925	0.087	2.523	0.875	7.275
1	Marital Status (no/yes)	-1.117	0.003	0.327	0.158	0.677
1	Children under 18 living at home? (no/yes)	− 0.091	0.903	0.913	0.209	3.979
1	Parents’ level of education? (university > 4 years as reference)		0.720			
	Primary school	0.647	0.444	1.910	0.365	9.998
	High School	0.945	0.273	2.573	0.475	13.956
	Higer Education up to 4 years	0.762	0.391	2.142	0.375	12.226
1	English skills (0–10)	0.238	0.034	1.269	1.019	1.582
1	Previous higher education (no/yes)	− 0.179	0.676	0.836	0.360	1.940
1	Origin (Nordic countries/other)	− 0.183	0.237	0.833	0.615	1.128
1	Previously exchange (no/yes)	1.581	0.002	4.792	1.738	13.214
1	Friends who have been on exchange (no/yes)	0.020	0.968	1.020	0.380	2.738
1	Perceived barriers to exchange (< 27, > 27)	− 0.126	0.742	0.882	0.418	1.862
1	Constant	-2.443	0.060	0.087		
9	Study program:		0.126			
	Nursing	1.552	0.035	4.721	1.117	19.947
	Paramedic	0.003	0.994	1.003	0.407	2.473
	Occupational therapy	0.636	0.190	1.890	0.730	4.890
9	Marital Status (no/yes))	-1.160	< 0.001	0.313	0.160	0.614
9	English skills (0–10)	0.248	0.015	1.282	1.050	1.565
9	Previously exchange (no/yes)	1.540	< 0.001	4.664	1.870	11.632
9	Constant	-2.135	0.015	0.118		

*Variable(s) entered on step 1: What is your age?, Sex, What study are you on?, What is your marital status?, Do you have children under 18 living at home?, What is your parents’ level of education?, English skills rated from 0–10, Previous higher education, Origin, Previously been on exchange?, Having friends who have been on exchange?, Perceived barriers to exchange, sum score, using a backward conditional elimination procedure

The Hosmer and Lemeshow goodness -of-fit-test: the Chi-square values range from 1.160 to 10.769 across the nine steps, suggests that the model fits the data reasonably well at each step. The p-values range from 0.215 to 0.992, further suggesting an acceptable fit of the model to the data at all steps.

### Qualitative results

There were 50 answers and 69 meaning units on the open-ended question of barriers for internationalization. Three overarching themes were developed: prerequisites for international exchange, personal barriers, and relations.

The first theme, Prerequisites for international exchange, consisted of nine categories representing issues related to everyday life of the student that would be affected if going abroad, and issues related to the universities’ demands and facilitation for exchange. Housing, both abroad and at home, was an issue the students were concerned about. Some reported not being able to travel because of obligations at work. Lack of information on exchanges from the university was reported to be a barrier. One student wrote “Lack of clarity in the professional difference between staying in Oslo and exchange”. Furthermore, practical issues like applying for visa are reported to be of importance. Also, language barriers were reported in the open-ended answers, like not being able to speak English. The demands for being accepted for exchange were also a barrier, as well as uncertainty of professional demands, admission to exchange, and lack of facilitation for the exchange to fit with the modules at home. The most reported barriers were related to financial issues, concerning lack of income while abroad, but also the extended costs of traveling and maybe dealing with costs both at home and at the exchange destination. One student directly reported “I do not have enough money to travel.”

The second theme, Personal barriers, consisted of five categories. The students reported the financial issues were of great importance as a barrier. They also reported a period of intense strain, with several tasks occurring concurrently, all in preparation for studying abroad. Another barrier reported by several students was health issues. Chronic diseases and fatigue were reported to be hard to combine with an exchange. Having placements “at home” was considered important to building the experience they needed to be considered for their preferred workplace when graduated. Some students of non-native Norwegian origin reported an importance of “learning about the Norwegian context”. The last category reported in the theme of personal barriers was related to a sense of safety, and insecurity at being away from family, friends or partner.

The third and last theme was Relations, where students reported barriers, both related to family relations and other social relations. Barriers in family relations were especially related to having small children to care for and being away from them over an extended period. Travelling without friends and being unsure of maybe having to spend a lot of time alone, was furthermore reported av relational barriers for exchange. As one student put it “I think it might be scary to travel without family nearby”.

## Discussion

The study engaged participants from four distinct professional health science bachelor programs. The sample was predominantly young and female, with the majority in their first year of their respective programs. A larger proportion of students had previous higher education experience, and most were single without children. When asked about their interest in study abroad programs, responses varied, with nearly half showing interest. The results indicated that those intending to participate in such programs were more informed about the process and had a greater desire to engage in both theoretical and clinical courses during the exchange. Perceived gains and barriers to participating in exchange programs were evaluated, with practical concerns like accommodation and loss of income rated as important barriers. Key factors associated with the intention to go on exchange included relationship status, English language skills, previous exchange experience, and being a nursing student. The higher odds of intending to participate in exchange programs among nursing students may be linked to the range of exchange opportunities in the program and a positive environment created by an approximately 20% annual exchange participation rate in the nursing program.

Qualitative data highlighted three main themes: prerequisites for international exchange, personal barriers, and relationships. These themes encompassed a range of elements, from practical issues to personal challenges and the importance of maintaining social connections while abroad. The findings indicate that social relationships play a critical role in the decision to participate or not participate in exchange programs. Social relationships can be defined as networks of individuals who are interconnected, thereby forming patterns and behaviors within their relationships [32]. The data shows that most students are single, and smaller proportion have children under age 18 years. Many students expressed difficulties in temporarily leaving their family and friends or cited responsibilities towards their children who are either dependent on them or are currently in school. Other students mention that they have responsibilities for pets, have a job, want to be close to friends, or fear being alone on exchange. These are practical and financial obligations that are not easy to solve, set aside, or take over for others for shorter or longer periods. In the study by De Winter, Van Mol and de Valk [33] being in a relationship is negatively correlated with going abroad. Here, women take more account of their partner’s wishes in their assessment than men do in their assessments about exchange. The same study shows that family relations can also play a subtle role in the choices the students make.

Other studies indicate that socioeconomic status is a driver for whether the student chooses to go abroad [34, 35]. Students with parents who have higher education, good finances, or who have been on exchange during their own studies or career, exchange more than other students who do not have this relational support. Our study`s findings suggest a similar pattern. As anticipated, students identified several prerequisites for international exchange, with secure housing both at home and abroad being an important factor. Housing scarcity poses a challenge, as students are hesitant to leave their secured accommodation due to strict subletting rules. Additionally, the potential loss of income during the exchange, rather than the fear of losing their current job, emerged as a concern for students who often work while studying.

The social networks students are part of, both within and outside the university, are also an important factor in the choices they make [34, 35]. The motivation to participate in exchange programs often arises from a combination of factors, including better job opportunities, improved educational prospects, wanderlust, and the search for new experiences [3, 5]. Here, finance is a key factor, leading many students to choose exchange program within ERASMUS which provides scholarships [36].

The socioeconomic situation of a student or their family is beyond the university’s control. However, our study reveals that students find the preparation and application processes too complex. To help overcome financial obstacles and other challenges, universities can actively promote available scholarship opportunities. Additionally, universities can streamline administrative processes to make exchanges simpler and more accessible.

To motivate and engage students in exchange programs, universities can also enhance social networks among students. Strengthening these networks should involve fellow students who have previously participated in exchange programs, as they can normalize the application process, improve students’ social connections within the institution, and more effectively enhance students’ intrinsic motivation compared to staff [34].

In a previously published study, Tavares [37] points out that international students in minority groups feel marginalized and excluded. Our study was conducted at a university where about 30% of the students are not ethnically Norwegian, and approximately 38% of the respondents in our study originated outside the Nordic countries. However, origin was not associated with the intention to participate in exchange programs. Although we do not have data on the number of non-Norwegians who apply for or participate in exchanges, they are in a significant minority. Tavares [37] therefore makes an important point in the effort to strengthen exchange within higher education: addressing structural inequalities between groups of students, irrespective of ethnic, social, or socio-economic background.

Available data from Erasmus plus for 2024 indicates a decrease in students participating in learning mobility compared to 2023, following a significant post-pandemic increase between 2021 and 2022. Current participation numbers are near pre-pandemic levels, suggesting a possible equilibrium. However, the EU budget for 2025 proposed a significant reduction to Erasmus plus (38, 39). At our university, over the last five years, the percentage of students going abroad for three months of their study program has varied between 5% and 20%, far below the government’s goal of 50% outgoing students.

This might indicate that future exchanges may increasingly take the form of shorter mobility programs (16), virtual exchange (40), or a combination of both, such as Blended Intensive Programs (BIP). For example, a BIP often includes an online component first, followed by a short stay abroad funded by ERASMUS PLUS or others, where students meet in international groups to finalize a project. Heymans et al (40) conclude that integrating Internationalization at Home activities such as virtual exchange into the curriculum can enhance intercultural competence among healthcare students, preparing them for different ways of knowing, being, and doing to ensure optimal healthcare. In shorter mobility programs, one or two teachers from each participating university often travel with the students and teach in the course. This arrangement facilitates easier follow-up, quick resolution of practical issues, and ensures coherent demands for all participating students (16).

Notably, some of our participants cited health issues and fatigue as significant obstacles to study abroad. To our knowledge, these concerns have not been reported in previous studies, highlighting a potential area for further research and consideration in the planning and implementation of exchange programs. Internationalization at Home activities may provide viable alternatives for these students. By combining online and brief international components, these programs offer the benefits of international exchange, such as intercultural competence development, without the same level of practical, physical or mental strain. Therefore, they may present a more accessible and manageable option for students who may not be able to participate in traditional exchange programs.

### Strength and limitations

The cross-sectional design of the study limits the ability to draw causal inferences from the data, as it represents a single point in time and does not capture changes in attitudes or behaviors over time. Additionally, while the sample size is sizable, it may not fully represent the diversity of experiences and perspectives within the student population. Furthermore, this study focuses specifically on health-related degrees, which may limit the generalizability of the findings to other disciplines. Other fields of study may yield different results regarding factors influencing students’ decisions to study internationally. These limitations should be considered when generalizing the findings to other contexts or institutions. The strengths of this study lie in its combination of both quantitative and qualitative survey data and the inclusion of students from four different bachelor programs. The study provides insights into the initial stages of the exchange process, focusing not only on those who choose to participate in exchange programs but also on those who opt out.

## Conclusion

This study provides new knowledge of barriers to participate in exchange programs post-pandemic. There are several personal and socioeconomic factors reported as barriers extending universities’ control, however, extending the informational part especially concerning finances and facilitation of the preparation process will be important barriers to break following this knowledge. In addition, exploring possibilities for international experiences available to all students, beyond relational, financial and health related barriers, will be of great importance in the future.

## Electronic supplementary material

Below is the link to the electronic supplementary material.

Supplementary Material 1

Supplementary Material 2

## Data Availability

The datasets used and/or analyzed during the current study are available from the corresponding author on reasonable request.

## Abbreviations

BIP: Blended Intensive Programs
COIL: Collaborative Online International Learning
HK-Dir: Norwegian Directorate for Higher Education and Skills
Sikt: Data Protection Official for Research

## References

[1] Wit Hd. Internationalization of higher education: the need for a more ethical and qualitative approach. J Int Students. 2020;10(1):i–iv.

[2] Fruhstorfer BH, Jenkins SP, Davies DA, Griffiths F. International short-term placements in health professions education—A meta‐narrative review. Med Educ. 2024;58(7):797–811. 38102955 10.1111/medu.15294

[3] Brown M, Boateng EA, Evans C. Should I stay or should I go? A systematic review of factors that influence healthcare students’ decisions around study abroad programmes. Nurse Educ Today. 2016;39:63–71. 27006034 10.1016/j.nedt.2015.12.024

[4] Tjoflåt I, Razaonandrianina J, Karlsen B, Hansen BS. Complementary knowledge sharing: experiences of nursing students participating in an educational exchange program between Madagascar and Norway. Nurse Educ Today. 2017;49:33–8. 27886624 10.1016/j.nedt.2016.11.011

[5] Blankvoort N, Kaelin VC, Poerbodipoero S, Guidetti S. Higher education students’ experiences of a short-term international programme: exploring cultural competency and professional development. Educational Res. 2019;61(3):356–70.

[6] Mashizume Y, Watanabe M, Fukase Y, Zenba Y, Takahashi K. Experiences within a cross-cultural academic exchange programme and impacts on personal and professional development. Br J Occup Therapy. 2020;83(12):741–51.

[7] Levitt O, Gilbert-Hunt S, Murray C, Baker A, Boshoff K. International allied health student placements: A meta-synthesis. Scand J Occup Ther. 2021;28(4):251–63. 32857632 10.1080/11038128.2020.1809703

[8] Hovland OJ, Johannessen B. Nursing students develop cultural competence during student exchanges in Tanzania. Norwegian Journal of Clinical Nursing/Sykepleien Forskning; 2019.

[9] Hovland OJ, Johannessen B. What impact did a student exchange have on participating nurses in the longer term? Nor J Clin Nursing/Sykepleien Forskning. 2021.

[10] Johnston J, McKenna L, Malik G, Reisenhofer S. Reported outcomes of nursing or midwifery students participating in international educational programs in their pre-registration education: A narrative systematic review. Nurse Educ Today. 2022;111:105320. 35276538 10.1016/j.nedt.2022.105320

[11] Wonson M, Boetto H, Moorhead B. Student participation in study abroad programs: social justice implications for tertiary education. Australian Social Work. 2021;74(1):83–95.

[12] Findlay A, King R, Stam A, Ruiz-Gelices E. Ever reluctant Europeans: the changing geographies of UK students studying and working abroad. Eur Urban Reg Stud. 2006;13(4):291–318.

[13] Vossensteyn JJ, Beerkens-Soo M, Beerkens M, Cremonini L, Besançon B, Focken N, et al. editors. Improving the participation in the Erasmus programme2013.

[14] Kosmaczewska J, Jameson S. Education first or tourism first - What influences the choice of location for international exchange students: evidence from Poland. J Hospitality Tourism Educ. 2023;35(2):143–58.

[15] Bønløkke M, van der Linde L. Intercultural educators exploring facilitation: A qualitative study on facilitating students’ learning process during a short exchange-AN ENM field study. 2021.

[16] Bjørnnes AK, Skaufel H, Holmström Rising M, Gädda M, Nieminen A-L, Misvær N, et al. Short-Term student exchange in nursing education: A descriptive pilot study. Sage Open. 2024;14(4):21582440241299522.

[17] Turale S, Kunaviktikul W, Mesukko J. Giving undergraduate nursing students international experiences: issues and strategies. Nurs Health Sci. 2020;22(3):830–6. 32277564 10.1111/nhs.12722

[18] Granel N, Leyva-Moral JM, Morris J, Šáteková L, Grosemans J, Bernabeu-Tamayo MD. Student’s satisfaction and intercultural competence development from a short study abroad programs: A multiple cross-sectional study. Nurse Educ Pract. 2021;50:102926. 33227616 10.1016/j.nepr.2020.102926

[19] Pereira HR, Brisbois MD, Silva HO, Stover CM. Learning beyond expectations: evaluation of an international nursing student exchange. J Nurs Educ Pract. 2018;8(2):72–82.

[20] Zadnik M, Psychouli P, Collins K. Developing an international cultural learning project: an effort towards introducing internationalization in the classroom. J Occup Therapy Educ. 2019.

[21] Heymans Y, Strosnider C, Pool J, van Jansen M. Fostering intercultural competence through virtual exchange: perspectives of undergraduate health students. Open Praxis. 2024;16(2):119–29.

[22] Hynes SM, Hills C, Orban K. A scoping review of online international student collaboration in occupational therapy education. Br J Occup Therapy. 2022;85:642–52. 10.1177/03080226221086221 PMC12033579 40336653

[23] Jenssen U, Bochenek JM, King TS, Steindal SA, Hestvold IV, Morrison-Beedy D. Impact of COIL: learning from student nurses in Norway who collaborated with U.S. students. J Transcult Nurs. 2023;35:74–82. 37933746 10.1177/10436596231209043 PMC10714699

[24] Kunnskapsdepartementet. (White Paper No. 7 (2020–2021) A World of Opportunities: International student mobility in higher education. In: Kunnskapsdepartementet, editor. 2020.

[25] de Wit H, Altbach PG. Internationalization in higher education: global trends and recommendations for its future. Policy Reviews High Educ. 2021;5(1):28–46.

[26] Sivertsen B, Johansen M. Studentenes helse- Og Trivselsundersøkelse 2022. Oslo: Studentsamskipnaden SiO; 2022.

[27] Sivertsen B, Knudsen AKS, Kirkøen B, Skogen JC, Lagerstrøm BO, Lønning K-J et al. Prevalence of mental disorders among Norwegian college and university students: a population-based cross-sectional analysis. Lancet Reg Health–Europe. 2023;34. 10.1016/j.lanepe.2023.100732 PMC10624983 37927428

[28] Kunnskapsdepartementet. Nasjonale Retningslinjer for helse- Og Sosialfagutdanningene (RETHOS). In: Kunnskapsdepartementet, editor. Oslo2019.

[29] Sikt.) NAfSSiEaR. [cited 2024 12/11]. Available from: https://sikt.no/en/home

[30] Hosmer DW Jr, Lemeshow S, Sturdivant RX. Applied logistic regression: Wiley; 2013.

[31] Graneheim UH, Lundman B. Qualitative content analysis in nursing research: concepts, procedures and measures to achieve trustworthiness. Nurse Educ Today. 2004;24(2):105–12. 14769454 10.1016/j.nedt.2003.10.001

[32] Bruggeman J. Social networks: an introduction. Routledge; 2013.

[33] De Winter T, Van Mol C, de Valk HA. International student mobility aspirations: the role of romantic relationships and academic motivation. J Stud Int Educ. 2021;25(5):505–23.

[34] Beech SE. International student mobility: the role of social networks. Social Cult Geogr. 2015;16(3):332–50.

[35] Haldimann L, Heers M, Rérat P. Youth on the move? The selectiveness of temporary mobilities from a life course perspective. Appl Mobilities. 2023;8(2):129–48.

[36] Deakin H. The drivers to Erasmus work placement mobility for UK students. Children’s Geographies. 2014;12(1):25–39.

[37] Tavares V. Feeling excluded: international students experience equity, diversity and inclusion. Int J Incl Educ. 2024;28(8):1551–68.

[38] EU programme for education t, youth and sport. Data visualisation on learning mobility projects [Available from: https://erasmus-plus.ec.europa.eu/resources-and-tools/factsheets-statistics-evaluations/statistics/data/learning-mobility-projects

[39] (ESN) ESN. Urgent Call to Preserve Erasmus + Funding 2024 [Available from: https://esn.org/news/urgent-call-preserve-erasmus-funding

[40] Heymans Y, Strosnider C, Pool J, Vuuren MJ. Fostering intercultural competence through virtual exchange: perspectives of undergraduate health students. Open Praxis. 2024;16(2):119–29.

