# Supplementary material for: Exploring intentions: factors influencing international study decisions in healthcare bachelor degree programs
Source: BMC Med Educ. 2025 Apr 17;25:555. doi: 10.1186/s12909-025-07136-4 (PMC12004624; doi:10.1186/s12909-025-07136-4)
Supplement: Supplementary file 1 — Supplementary Material 1 [file 12909_2025_7136_MOESM1_ESM.pdf]

**Supplemental File Study Questionnaire**

**Section 1: Participant Demographics**

1. What is your gender?

  - [ ] Woman
  - [ ] Man
  - [ ] Other
  - [ ] Prefer not to disclose

2. What is your age group?

  - [ ] < 22 years
  - [ ] 22-25 years
  - [ ] 26-30 years
  - [ ] > 30 years

3. What is your current field of study?

  - [ ] Nursing
  - [ ] Occupational Therapy
  - [ ] Learning Disability Nurse
  - [ ] Paramedic

4. Which year of study are you currently in?

  - [ ] Year 1
  - [ ] Year 2
  - [ ] Year 3

5. Have you pursued any previous higher education?

  - [ ] Yes
  - [ ] No

6. What is your marital status?

- [ ] Single
- [ ] Married/Domestic Partnership

7. Do you have children under 18 years old?

- [ ] Yes
- [ ] No

8. What is the highest level of education attained by your parents?

- [ ] Primary
- [ ] High school
- [ ] University (<4 years)
- [ ] University (>4 years)

9. What is your origin?

- [ ] Nordic
- [ ] Europe
- [ ] Africa
- [ ] Asia
- [ ] Oceania
- [ ] America

10. Have you ever participated in an exchange program before?

- [ ] Yes
- [ ] No

11. Do you have friends who have participated in an exchange program?

- [ ] Yes
- [ ] No

12. Do you want to go on exchange during your program?

- [ ] Yes
- [ ] No
- [ ] Unsure

Please elaborate here:

____________________________________________________________

This element is only displayed if the option "Yes or Unsure" is selected in the question "Do you want to go on exchange?"

**Section 2: Knowledge about Exchange Programs**

12. Do you have knowledge about exchange programs in your program?

- [ ] Yes
- [ ] No
- [ ] Unsure

13. Do you know when you can participate in an exchange program?

- [ ] Yes
- [ ] No
- [ ] Unsure

14. Are you aware of the destinations available for exchange?

- [ ] Yes
- [ ] No
- [ ] Unsure

15. Would you like to participate in an exchange program for theoretical courses?

- [ ] Yes
- [ ] No
- [ ] Unsure

16. Would you like to participate in an exchange program for clinical courses?

- [ ] Yes
- [ ] No
- [ ] Unsure

17. Is your preferred exchange destination offered in your program?
    - [ ] Yes
    - [ ] No
    - [ ] Unsure

18. Do you know about ERASMUS support for exchange programs?
    - [ ] Yes
    - [ ] No
    - [ ] Unsure

19. Do you know about Lånekassen support for exchange programs?
    - [ ] Yes
    - [ ] No
    - [ ] Unsure

20. Do you know about other support sources for exchange programs?
    - [ ] Yes
    - [ ] No
    - [ ] Unsure

21. Has any of your courses been taught in English?
    - [ ] Yes
    - [ ] No
    - [ ] Unsure

22. Please rate your English skills on a scale from 0 to 10.

- [ ] (Scale: 0-10)

**Section 3: Intention and Perceived Gains from Exchange Programs**

23. Rate the following perceived gains from an exchange program on a scale of 1 to 5:

- Learning about communication
  - [ ] 1 (Strongly Disagree)
  - [ ] 2 (Disagree)
  - [ ] 3 (Neither Agree nor Disagree)
  - [ ] 4 (Agree)
  - [ ] 5 (Strongly Agree)

- Learning about culture
  - [ ] 1 (Strongly Disagree)
  - [ ] 2 (Disagree)
  - [ ] 3 (Neither Agree nor Disagree)
  - [ ] 4 (Agree)
  - [ ] 5 (Strongly Agree)

- Improve language skills
  - [ ] 1 (Strongly Disagree)
  - [ ] 2 (Disagree)
  - [ ] 3 (Neither Agree nor Disagree)
  - [ ] 4 (Agree)
  - [ ] 5 (Strongly Agree)

- Learning new skills
  - [ ] 1 (Strongly Disagree)
  - [ ] 2 (Disagree)
  - [ ] 3 (Neither Agree nor Disagree)
  - [ ] 4 (Agree)

- [ ] 5 (Strongly Agree)

- Improved creative skills
  - [ ] 1 (Strongly Disagree)
  - [ ] 2 (Disagree)
  - [ ] 3 (Neither Agree nor Disagree)
  - [ ] 4 (Agree)
  - [ ] 5 (Strongly Agree)

- Improved flexibility
  - [ ] 1 (Strongly Disagree)
  - [ ] 2 (Disagree)
  - [ ] 3 (Neither Agree nor Disagree)
  - [ ] 4 (Agree)
  - [ ] 5 (Strongly Agree)

**Section 4:** Perceived Barriers to Exchange

24. Please indicate the importance of each barrier to you when considering going on an exchange program on a scale of 1 to 5:

- How important is the concern of renting out your apartment while you are on exchange?
  - [ ] 1 (Not at all important)
  - [ ] 2 (Slightly important)
  - [ ] 3 (Neutral)
  - [ ] 4 (Moderately important)
  - [ ] 5 (Very important)

- How important is the concern of potentially losing your job if you go on exchange?
  - [ ] 1 (Not at all important)
  - [ ] 2 (Slightly important)
  - [ ] 3 (Neutral)

- [ ] 4 (Moderately important)
    - [ ] 5 (Very important)

- How important is the concern of losing income while you are on exchange?
    - [ ] 1 (Not at all important)
    - [ ] 2 (Slightly important)
    - [ ] 3 (Neutral)
    - [ ] 4 (Moderately important)
    - [ ] 5 (Very important)

- How important is it for you to have a clinical placement in Norway for your summer work?
    - [ ] 1 (Not at all important)
    - [ ] 2 (Slightly important)
    - [ ] 3 (Neutral)
    - [ ] 4 (Moderately important)
    - [ ] 5 (Very important)

- How important is it for you to have a clinical placement in Norway for your post-graduate work?
    - [ ] 1 (Not at all important)
    - [ ] 2 (Slightly important)
    - [ ] 3 (Neutral)
    - [ ] 4 (Moderately important)
    - [ ] 5 (Very important)

- How important is the concern of facing language barriers while on exchange?
    - [ ] 1 (Not at all important)
    - [ ] 2 (Slightly important)
    - [ ] 3 (Neutral)
    - [ ] 4 (Moderately important)
    - [ ] 5 (Very important)

- How important is the concern of having too much work to prepare for the exchange?

    - [ ] 1 (Not at all important)

    - [ ] 2 (Slightly important)

    - [ ] 3 (Neutral)

    - [ ] 4 (Moderately important)

    - [ ] 5 (Very important)

- How important is the concern of the application process being too extensive?

    - [ ] 1 (Not at all important)

    - [ ] 2 (Slightly important)

    - [ ] 3 (Neutral)

    - [ ] 4 (Moderately important)

    - [ ] 5 (Very important)

25. Do you experience any other barriers to going on exchange? Please elaborate here:

______________________________________________________________

Thank you for completing this questionnaire.
